# Supplementary material for: Are carbon emissions trading and green financial instruments synergistic? -Comprehensive quantitative research based on content analysis
Source: PLoS One. 2024 Mar 7;19(3):e0298601. doi: 10.1371/journal.pone.0298601 (PMC10919601; doi:10.1371/journal.pone.0298601)
Supplement: S1 Data — (ZIP) [file pone.0298601.s001.zip › Data/地级市Z_F工作报告.zip!/║┌┴·╜¡/╥┴┤║╩╨/└┤╫╘╣1⁄2╓┌║┼í╛┬φ┐╦╩2╛▌═°í┐_2010.docx]

各位代表：

现在，我代表市政府，向大会作工作报告，请予审议，并请政协委员和列席会议的同志提出意见。

2009年的主要工作

2009年，是新世纪以来我市极不寻常、极不平凡的一年，是受外部冲击最大、困难最多、挑战最为严峻的一年，也是全市上下迎难而上、共克时艰、夺取全面胜利的一年。在备受金融危机困扰，又遭遇春季过界山火、夏季连天阴雨的极端复杂情况下，我们紧紧依靠省委、省政府和市委的坚强领导，团结带领全市人民坚持不懈保生态，千方百计促增长，尽心竭力惠民生，圆满地完成了市十二届四次人代会确定的各项任务。

——有力应对金融危机，主要经济指标逆势增长。年初，金融危机给我市支柱产业的“钢木经济”带来严重冲击，形势之严峻多年罕见。西钢及全市绝大多数木材加工企业陷入亏损、停产状态，其它产业也不同程度地受到影响。面对困境，我们科学分析研判形势，及时果断地打出了应对“组合拳”。针对重点骨干企业效益下滑的问题，出台了《帮助企业保增长促发展的意见》和《扭亏增盈工作实施方案》等一系列政策措施，深入开展了“百日攻坚保增长”活动，使工业由一季度的持续亏损转为二季度以后的逐月回升。全年规模以上工业企业实现利润4.7亿元，同比增长22.3%。针对上半年木材市场疲软的问题，采取制定最低保护价、统一竞价等措施，使木材销售价格、任务达到了预期目标。针对大豆、玉米、木耳等农副产品滞销的问题，采取争取国家增加保护价收购配额、多方促销等措施，切实保护了农民和职工利益。针对企业融资和大项目建设配套资金困难等问题，采取协调银企对接、举办金融论坛、增加担保资本金注入、成立小额贷款公司、发行企业债券等措施，缓解了资金压力。针对林产工业企业出口受阻、停产企业增多等问题，积极组织企业依托光明等大企业转向开发国内市场，扭转了被动局面。针对一些企业裁员问题，及时拨付7600万元失业保险金支持企业，稳定了4.5万人的就业岗位。在救企业、保就业的同时，我们主动化危为机，积极与国家扩内需政策对接，全年共争取到保障性安居工程、农村工程和重大基础设施、医疗卫生、教育文化、节能减排和生态建设、重点产业振兴及技术改造等方面项目287个，争取到国家和省投资15.1亿元，比2008年增长了2.1倍，争取到国家投资占全省的10.1%，争取省配套投资占地市总额的18.5%。成功发行了12亿元债券，是全省第二个争取到发行企业债券的地市。年初确定的23个重点项目，有7项被列入省200个重点大项目之中。可以说，2009年是伊春有史以来对上争取项目投资力度最大、成效最显著的一年。在诸多有力的政策措施支撑推动下，全年经济实现了由回落、企稳回升到加快发展的向好转变。全市生产总值预计实现171.3亿元，同比增长12.6%，连续六年保持两位数增长；全口径财政收入实现14.7亿元，同比增长32.4%，其中地方财政收入实现8.8亿元，同比增长47%；城镇居民人均可支配收入实现9453元，同比增长8.4%；农民人均纯收入实现6139元，同比增长14.3%。

——全力保护资源环境，生态功能区建设扎实推进。坚持系统化推进生态功能区建设，认真落实“严管林”措施，狠抓防伐、防垦和防火工作。依法收回了农林交错地带被侵权发证的林地1500多亩;实施整理土地项目11个，新增耕地1320公顷；完成水土流失治理1620公顷。全年造林绿化20.3万亩，防治有害生物面积145万亩。持续加大对红松、“三大硬阔”、蓝莓、林蛙等珍贵物种的保护力度，累计3.4万人参与认领红松8.03万株，募集红松保护基金803万元，比上年增加191万元。新青的国家级白头鹤自然保护区通过评审，全市各级各类自然保护区总数已达到21个70.3万公顷。新青获得“中国白头鹤之乡”称誉。红星大平台、嘉荫茅兰沟、桃山悬羊峰合并晋升为小兴安岭国家地质公园。森林防火能力建设进一步加强，基础设施明显改善，红星、新青等北部重点火险区“天眼工程”试点取得阶段性成果，成功扑灭了“4·27”沾河过界森林大火，创造了我市依靠人力扑灭最大面积山火的纪录。积极推进节能减排，实施化学耗氧量减排项目7个，集中饮用水源地水质和二级空气质量达标率分别达到100%和98.9%。以美溪、朗乡、乌马河为示范的林场所撤并改造和烧柴改革试点顺利启动，全市第一阶段撤并30个林场所的目标，随着今年棚改户的回迁将全面完成。发起并承办了首届东北亚生态论坛，举办了中国环境与健康发展论坛，并被确定为这两个论坛的永久举办地。对外发布了首个年度生态建设报告，我市的生态建设在国内外的知名度和影响力进一步扩大。

——努力调整经济结构，经济转型取得重大进展。五大接续替代产业预计实现增加值65.5亿元，同比增长15.5%，占GDP比重同比增加0.6个百分点。农业生产战胜严重低温、内涝等自然灾害获得丰收，实现增加值51.7亿元，同比增长11.4%。粮食总产达到69.2万吨，同比增长8%；畜牧业实现增加值9.8亿元，同比增长18.4%；农业产业化经营水平不断提升，规模以上加工企业达到50户，其中，省级产业化重点龙头企业达到8户；铁力市被确定为省级农技推广体系改革示范县。积极发展县域经济。铁力市与森工、农垦企业密切合作，增强了区域经济加快发展的合力；嘉荫场县共建取得重大突破，实现土地流转4.85万亩、代耕76万亩。工业经济迈上新台阶。规模以上工业实现增加值35.7亿元，同比增长20%，高于GDP增幅7.4个百分点。冶金、建材化工、森林食品加工、木材精深加工等基地载体作用明显，工业企业完成投资34.1亿元，同比增长7.7%，其中亿元以上项目投资占工业投资的48.5％。西钢烧结机改造、嘉荫亿通木业实木复合地板、金山屯大白山少白山风电场等项目相继投产。特别是西钢集团在国内近半数大型钢铁企业仍在亏损运行的情况下，5月份冲出重围实现盈利，全年本部钢产量达到191.2万吨，为我市保增长做出了重要贡献。第三产业实现新跨越。预计实现增加值54.9亿元，同比增长9.5%。接待游客382万人次，旅游业实现收入19.1亿元，同比分别增长13.5%和26.7%。特别是冬季旅游实现了历史性突破，仅12月份游客就达到18.5万人。我市成为各国驻华大使“最值得向世界推荐的中国生态旅游城市”，汤旺河国家公园被评为“中国十佳生态旅游示范基地”，铁力市被评为“中国优秀生态旅游城市”。商贸流通、交通运输、社区服务、保险等服务业活力增强，社会消费品零售总额实现48亿元，同比增长18.8%。金融、税务部门对经济社会发展的支持力度加大，全市金融机构发放各项贷款余额达86.4亿元，比年初增加9.1亿元。非公有制经济得到了新发展，实现增加值92.2亿元，同比增长14%。科技创新步伐加快。组织实施各级各类项目93项，科技型企业发展到88户，13户高新技术企业实现产值57.2亿元，同比增长21%。

——着力深化改革开放，发展活力进一步增强。国有林权制度改革试点深入推进，破解了权属证发放与采伐指标争取等难题。国企改革迈出新步伐。光明集团继摘星保壳之后，强化新产品研发和市场开拓，实现了扭亏为盈，并带动了19户家具企业联合发展；伊春啤酒公司被华润雪花啤酒有限公司成功收购，5万千升啤酒技改工程投产；晨明水泥厂、带岭造纸厂政策性破产司法终结,浩水公司对晨明水泥厂、美溪建材公司实现了重组，一批多年的停产半停产企业和闲置资产被盘活，成为经济的新增长点。以项目招商为重点，全面推进对外开放。全年共引进国内投资122项，到位资金56.7亿元，同比增长10.7%；实际利用外资2769万美元，同比增长85%；外贸进出口总额完成1.92亿美元。对俄经贸合作取得新成效，嘉荫口岸过境首次突破万人大关，达到1.56万余人次。嘉荫海关和黑龙江出入境检验检疫机构进驻市中心区，为企业办理出口手续打开了方便之门。

——强力推进基础设施建设，城镇载体功能显著提升。2009年是我市固定资产投资最多、基础设施项目建设最多的一年。固定资产投资首次突破百亿，同比增长47.6％，增幅高于全省7.6个百分点。林都机场通航运营，兴安湖水库下闸蓄水，伊绥高速和沿边公路、华能热电等一批事关伊春长远发展、承载几代林区人期盼与梦想的重大基础设施项目正在加紧建设。全年共完成城市基础设施建设投资3.5亿元，新建改造道路25.1万平方米、铺装人行道板6.4万平方米、维修改造直管楼房140栋、楼房平改坡3.7万平方米。中心城和南岔区等地的污水处理厂建成投产，林业发电厂热源建设项目按期竣工，全市供水、供热普及率各提高4个百分点，分别达到64%和50%。植树101万株，新增绿化面积263.8公顷，覆盖率增加2.36个百分点。严格规范土地市场，实现土地资产收益2.27亿元，是上年的3.57倍。房地产市场日趋活跃，全年住房销售84.34万平方米，为上年的1.6倍。第二轮新农村建设试点全面展开，完成农村公路114条961.4公里，通乡、通村公路硬化率分别达到100%和98.6%。农村人均住房建筑面积达到19.61平方米，住房砖瓦化率达到81.95%；建设农村饮水安全工程54处，新打抗旱井579眼，改善水田灌溉面积3.32万亩。

——竭力保障和改善民生，发展成果惠及全市人民。2009年，是全市人民得到实惠最多的一年。我们以前所未有的力度加大了民生投入，在全年51.3亿元的一般性财政支出中民生占比达到50%。拆迁棚户区180万平方米，开工建设272万平方米；改造农村泥草房2206户19.7万平方米；建设廉租房7209套36万平方米。城镇居民人均住房建筑面积达到21.41平方米，比上年增加0.81平方米。27.4万城镇居民纳入了医疗保险，社区卫生服务人口覆盖率达到85%以上，新农合覆盖率达到100%。甲流疫情得到有效防控。中心区公交车辆更新90台，线路延伸到了翠峦区; 新开通农村客运线路7条，进一步方便了群众出行。建成1000平方米以上的标准化社区服务中心16个。全部兑现了机关事业单位人员调资和公务员津贴补贴，分别为政企合一区及森工企业在职人员、企业退休人员月人均增资114元和147元，为8.4万退休人员补发历史拖欠项目内养老金1.4亿元，为4.2万“五七工”、“家属工”解决了养老保险问题，14.8万困难群众享受到最低生活保障。第四轮扶贫解困任务全面完成。实现就业再就业4.7万人，城镇新增就业3.9万人，登记失业率为4.3%，低于省控指标0.3个百分点。千方百计引导和支持全民创业，全年共培训创业者5000多人，发放小额贷款1012万元。积极促进教育公平，全面实施义务教育学校标准化建设和校舍安全工程，推进素质教育，全市基础教育质量提前一年实现了“打翻身仗”的目标，职业教育得到加强。组织开展了“城市之光”、“大森林之声”系列文化和全民健身活动，我市运动员在参加国内外比赛中获得奖牌263枚。“四城联创”扎实推进。经过全市上下的共同努力，市政府年初做出的为民办实事承诺全部兑现！

——大力加强民主法制建设，社会局面更加和谐稳定。主动向人大及其常委会报告工作，虚心听取政协的协商意见，认真办理提案、议案，自觉接受人大代表、政协委员及全市人民的监督。建立了政府领导与网民在线交流制度，及时处理了群众提出的问题。强化依法行政，推行行政执法责任制和行政问责制度，切实强化了规范权力运行的制度建设。全面开展了“六型”政府机关创建、“查、找、比”和“百企万民评服务”等活动，大力推行政务公开，认真清理各种规费，全面清理行政审批事项，强化了电子政务建设。政府机关作风建设得到加强，行政效能有所提高。“平安伊春”建设取得积极成效，社会治安防控体系不断完善，信访、安全生产和食品药品安全及质量监督工作进一步加强，社会大局持续稳定。计划生育、妇女儿童、国防人防、民族宗教、统计审计、外事侨务、史志档案、防灾减灾和老龄、双拥等工作也取得了新成绩。

各位代表、同志们，一年的成绩来之不易。这得益于省委、省政府和市委的正确领导，得益于市人大、市政协和社会各界的戮力同心、鼎力相助，得益于我们对形势的科学研判和对发展策略的准确把握，得益于全市上下振奋精神、众志成城、奋力攻坚！在此，我代表市人民政府，向辛勤工作在各条战线上的全市人民和关心支持伊春建设的各位领导、朋友们致以崇高的敬意和衷心的感谢！

各位代表、同志们，过去的一年，虽然我市取得了一些成绩，但按照科学发展观的要求，与走在我们前面的地区相比，用人民群众的期盼衡量，还存在一些突出矛盾和问题。主要是：长期形成的体制性、机制性、结构性和社会性等深层次矛盾和问题还没有彻底解决；接续替代产业发展慢，企业融资渠道仍然不畅，特别是附加值和税收较高的企业和项目太少，财税增长后劲乏力，加之财政供养人口较多，老龄化提早到来，养老、医疗等负担过重，“小财政”支撑“大民生”的矛盾比较突出；有的政府部门思想解放不够、职能转变滞后，个别工作人员责任意识、服务意识、发展意识不强，工作作风不实、效率不高，谋项目、抓招商的动力不足，以权谋私、刁难勒卡的现象依然存在。对此，在今后的工作中，我们必须认真加以解决。

2010年的形势和任务

今年是“十一五”规划的收官之年，也是国家实施大小兴安岭生态保护与经济转型规划的开局之年。做好今年的工作，对加快我市的经济社会发展、为“十二五”发展奠定良好基础具有十分重要的意义。科学研判和准确把握形势是做好今年工作的基础。一方面，国际金融危机对我市发展的影响仍未消除，经济回升向好的基础还不牢固，我们既要解决长期累积的矛盾，也要做好应对短期市场波动的准备，特别是从根本上转变发展方式、推动经济转型还需要我们付出艰苦的努力。另一方面，我们也面临着诸多有利条件：一是宏观形势比较有利。我国经济已经率先在全球呈现“V”型反转，今年国家继续实施积极的财政政策和适度宽松的货币政策，投资仍将保持较大规模，并将着力提高政策的针对性和灵活性，特别是通过产业和区域“共振”的布局来拉动经济、调整结构，必将催生新的投资机会，再加上国际经济走向复苏，出口市场预期转好，有利于我市加快结构调整，加快转型升级，也有利于我市企业开拓国内外市场。二是支持政策逐渐增多。随着国家《进一步实施东北地区等老工业基地振兴战略的若干意见》的落实和天保工程的延伸，以及国家大小兴安岭生态保护与经济转型规划的出台，我市加快生态建设、发展生态产业以及政府财政都会得到新的支持，也有助于我们争取投入解决基础设施和民生的欠账。三是发展潜力开始显现。全市生态建设基础日渐巩固，发展思路日趋完善，经济转型步伐明显加快，民生实惠逐年增多，特别是全市人民思变、思进、思富的愿望更加强烈，思想解放程度、招商引资力度、谋划项目广度显著增强，来伊春的投资者也明显增加，这些都为我市实现跨越式发展奠定了坚实基础。只要我们把握好政策机遇、招商机遇、发展机遇，把全市人民的发展激情保护好、引导好，乘势而为，开拓进取，励精图治，周密谋划，扎实工作，就一定能够推动全市各项事业取得更大的进展。

各位代表、同志们，今年市政府工作的总体要求是：认真贯彻党的十七大、十七届三中、四中全会、中央和省委经济工作会议、市委十届十次全体（扩大）会议精神，以邓小平理论和“三个代表”重要思想为指导，深入贯彻落实科学发展观，解放思想，开拓奋进，牢牢把握“在保护中加快发展，在发展中兴市富民”的主基调，深入实施“生态立市、产业兴市”战略，突出把握政策机遇，突出抓好招商引资，突出引导全民创业，着力巩固扩大生态保护成果，着力转变发展方式优化产业结构，着力保障和改善民生，着力完善城市整体功能，推动全市经济社会又好又快、更好更快发展。

综合考虑各方面因素，今年全市的主要预期目标是：生产总值增长11%，规模以上工业增加值增长14%，地方财政一般预算收入按可比口径增长11%，全社会固定资产投资增长70%，社会消费品零售总额增长12%，国内引资实际到位额较上年计划增长20%，外贸进出口总额增长15%，实际利用外资与上年计划持平，城镇居民人均可支配收入增长8%，农民人均纯收入增长6%，城镇登记失业率控制在4.6%以内。

围绕上述总体要求和目标，须着力推进八项重点工作。

一、进一步强化资源保护和节能减排，加快生态功能区建设

丰富的自然资源和良好的生态环境是我市实现可持续发展的宝贵条件和重要依托，保护好这方资源和生态是伊春人世世代代要承担的神圣使命，我们一定要将这项工作进行到底。全力争取停止主伐。抢抓世界气候大会后国家实施控制温室气体排放行动和实施大小兴安岭生态保护与经济转型规划的历史性机遇，积极对上争取生态补偿，争取今年停止森林主伐，并确保停伐后软着陆、无震荡。不断加强资源培育和保护。继续实施天保工程，强化科学造林、质量兴林，重点搞好铁力等林业局森林经营和抚育试点工作，探索恢复森林数量和质量的有效途径。积极调整林分结构，加大“老三松”、“三大硬阔”等珍贵树种、经济林树种的营造比例，红松、混交林营造比例分别达到25%和30%以上，全年造林绿化28万亩，营建改培红松果林6500亩。抓好种苗产业的发展，加快苗木繁育基地建设。继续强化对红松、蓝莓、林蛙等珍贵物种的保护和恢复，深入推进自然保护区建设，保持和加快恢复生物多样性。继续强化森防能力建设，严格落实森防工作责任，创新森防技术与扑火机制，严防发生重大森林火灾。加快实施林场所撤并。抓住林区棚户区改造的政策机遇，大力实施生态移民，巩固好去年撤并30个林场所的成果，今年力争再撤并20个。强力推进烧柴改革。积极转变生产生活方式，大力推广地源热泵、沼气应用和生物质气化、半气化等替代燃料节能技术，将烧柴改革推广到所有中心林场所，减少对林木资源的消耗。深入抓好节能减排。继续推进重点减排工程建设，深入抓好钢铁、化工、建材等重点耗能企业的节能监察，加快推进建筑节能改造工程。强化技术支撑，大力发展循环经济、低碳经济。积极推进西林、朗乡等循环经济园区建设，支持浩水、浩化合作打造浩良河建材化工循环经济园区，加快发展粉煤灰、废旧物资再利用、生活垃圾焚烧发电等循环经济产业链。强化对矿产资源的保护与整合，做到有序、规模化开发。深入开展“保护母亲河—汤旺河”行动，全面完成松花江规划治污项目，抓好农药、化肥残留物等污染物的综合整治，倡导绿色消费，减少面源污染，确保完成“十一五”节能减排目标。

二、坚定不移地走新型工业化道路，做大做强优势工业

抓住国家和省实施产业调整振兴规划以及支持资源枯竭城市经济转型的政策机遇，适应沿海地区产业向内地转移辐射的趋势，加快接续替代产业发展，全力推进“4111工程”。深入实施大项目牵动战略。进一步研究国家扩内需政策，结合我市五大产业和四大基地建设需要，有针对性地加快谋划一批具有区域特色、产业特色、突出集聚发展的大项目，增加项目储备。积极推进惠天力日处理1000吨鲜奶、升辉集团越橘庄园酒堡等项目建设，争取500千伏输变电工程、铁力鹿鸣钼矿开发等市级重点产业项目早日开工。进一步延长西钢、浩水、浩化等企业的生产链条，支持西钢分离延伸上下游项目，上马1260高炉，分离上马制氧与回转窑和80万吨球团等团组项目，推进浩水日产5000吨水泥熟料生产线建设，尽快做大产能。加速朗乡木塑生产线、西林铅冶炼项目建设，尽快形成新的经济增长点。加快工业结构优化升级。通过资源整合、要素重组，抓好基地和园区建设，促进产业集聚发展。大力发展绿色能源业。重点抓好带岭东山、新青长青山、桃山马鞍山风电以及上甘岭红山和朗乡半圆河水电站建设，抓好兴安新能源公司太阳能电池片生产，打造一批技术含量高、市场竞争力强、经济效益好的省级绿色能源领军企业。提升木材精深加工业。强力推进林产工业兼并重组，搞好专业分工，实现上下游企业联名联牌发展。以光明家具为龙头，依托中心区和双丰，继续整合家具制造业，形成产业规模；以龙乡板业为龙头，组建跨区人造板集团，整合人造板企业。推进家具、人造板、小木制品企业开发科技含量高、附加值大的终端产品，推动林产工业品牌化、集团化。加快原料林基地建设，推进“林板一体化”进程。搞好市生态产业园区建设，最大限度地降低门槛，吸引更多的生态项目入驻园区，形成孵化、聚集效应。加快科技进步和自主创新步伐，鼓励和支持企业建立相关产业战略联盟，通过引进消化吸收新技术，培育自主创新能力，不断促进产品升级换代，推动产品向高端发展。抓好工业经济运行。加强对重点企业在生产运营、运输需求、产品价格等方面的运行监控分析与协调服务，继续开展“送政策、送服务、送温暖”活动，认真落实帮扶措施，引导和帮助企业调整经营策略和产品结构，提升应变力和竞争力，增强发展后劲。

三、全面落实惠农支农政策，实现农业增产、农民增收、农村繁荣

加大政策支持，优化产业结构，推进农业农村经济加快发展。努力提高农产品产能。按照稳定总产、提高单产、优质高效的要求，抓好粮食高产创建活动，大力发展设施农业、特色农业，生产绿色、有机、无公害农作物。继续实施“八十万头生猪”、“十五万吨鲜奶”战略工程，稳步抓好生猪、奶牛、肉牛基地建设和野猪、鹿等特色养殖，提高产业化水平。大力发展森林食品产业。认真落实《发展森林食品产业的意见》，着力发展食用菌、山野果、山野菜、驯养动物食品、野生保健食品、矿泉水等六大系列产品。抓好双丰五味子、乌伊岭榛子林、友好野猪驯养等保护型、改培型、培育型和野化型基地建设。重点推进以黑木耳为主的食用菌产业素质提升，力争在品种结构、原料替代、技术推广、产品加工、市场营销等方面实现新突破。扶持升辉、忠芝、银色食品、葵花药业等龙头企业加快发展，延长产业链，提高产品附加值，扩大市场占有率。通过嫁接引资和政策引资，吸引国内外有影响的食品、药品加工企业在我市落户。调整种植业结构，扩大适宜林区条件的北药、蓝莓等经济作物种植规模。进一步加强对蓝莓等珍贵物种的保护和人工培育，重点抓好五营等地组培蓝莓苗木繁育推广中心建设。发挥好林特产品检验中心的功能，搞好以有机食品为主的产品认证，强化产品质量，打造伊春森林食品品牌。继续加强农业基础设施建设。加快灌区、节水改造、病险水库加固等农田水利设施建设，落实农民购置大型农机具和深松整地的补贴政策，积极发展农机作业合作社，推广先进的农机耕作技术。全力抓好现代农业科技示范园区和农业信息化示范村建设。加强农业气象灾害监测预警，提高防灾减灾能力。深入开展局场县共建。支持铁力市与森工、农垦合作，完善“1+5”区域经济发展模式，促进区域之间构建优势互补、利益共享、相互带动的发展新格局。支持嘉荫创新共建方式，拓展合作领域，完成土地流转10万亩，鼓励农民到境外承包耕种土地，拓宽农民致富门路。扎实推进第二批新农村试点建设。加大帮建力度，统筹发挥政府主导和群众主体作用，抓好农村路、水、能、房等基础设施建设，加快各项社会事业发展，努力提高农民和务农职工的综合素质，不断提升我市农村和林场所整体发展水平，全面完成第二批试点任务。

四、加快发展以旅游业为主导的现代服务业，发挥好对经济、社会、文化发展的拉动作用

鼓励、支持服务业提档升级，积极培育新的增长点。全力推进生态旅游业。继续整合旅游资源，按照“提升夏季、突破冬季、拓展春秋”的思路，将夏季旅游向养生度假游延伸，将冬季旅游向体验游突破，将春秋旅游向会展游、休闲体育游拓展。着力加快汤旺河国家公园、桃山国际狩猎场、五营国家森林公园、嘉荫恐龙国家地质公园和小兴安岭国家地质公园等景区建设，努力打造国家和世界级的旅游精品。抓好乌马河西岭生态园、梅花山滑雪场和铁力国家动植物园、日月峡滑雪场等旅游项目建设，完善配套设施，提升档次水平。搞好中心区、铁力、嘉荫等地的中高档宾馆、家庭旅馆、休闲体育和娱乐设施建设，全力提高服务质量和接待能力。年内确保新开工建设三家以上五星级宾馆，改善旅游城市软环境。以游客生活消费习惯为导向，引导商业网点和公共服务行业调整营业时间，创新服务模式，把城市运行方式转到旅游城市的轨道上来。大力开拓客源市场，在巩固哈尔滨、京津等北方旅游市场的基础上，加大长三角、珠三角地区和西部省份的旅游市场开发力度，积极与国内知名旅行社合作开展包机、包专列旅游服务。着力发挥好旅游业对房地产、森林食品、木制工艺品和文体产业的带动作用，促进产业联动，助推旅游业可持续发展。加快发展会展业。探索市场化办节方式，办好杜鹃花观赏节、森林生态旅游节、兴安蓝莓节、五花山观赏节、冬季摄影节和环境与健康论坛等节事活动，筹划举办中国首届木制工艺品博览会，提高会展经济综合效益。加强与国内外会展名城以及著名展览组织的合作与交流，争取在更大范围内实现与国内外会展业对接，提升会展经济的层次。优化升级商贸流通业。加快物流平台建设，以伊春东北亚物流、光明、邮政物流为主渠道，以配货站和个体运输为补充，不断壮大物流体系。抓好兴安岭森林产品大市场等市场建设，将我市打造成全国知名的森林产品集散地。继续实施“万村千乡”市场工程，开展“家电下乡”活动，加强供销网络建设，实现村屯、林场所供销连锁网络全覆盖。加快信息技术开发与应用，整体推进信息化建设，提升林区现代化水平。

五、继续深化改革开放，增强经济发展活力和动力

抓住影响和制约发展的关键环节，全力破解体制性障碍，抓好重点领域改革，扩大对外开放，建立完善有利于保增长、促发展的体制机制。争取扩大林权制度改革。继续抓好林改扩面的前期准备工作，在获得国家批准后及时启动实施。进一步规范、管理好承包林地内的采伐经营活动，继续引导、扶持承包户大力发展林下经济，搞活林地经营。逐步完善配套改革，探索解决好活立木交易市场启动、融资和林木参保等关键性问题，推进林改向纵深发展。进一步深化国企改革。认真处理好带岭造纸厂、晨明水泥厂破产后的遗留问题，推进浩水公司重组晨明水泥厂、美溪建材公司后尽快投达产。大力支持光明集团股改重组，实现资产的有效整合。加快升辉集团上市步伐，同时抓紧培育其它有条件的企业上市。认真搞好政府机构改革。按照省政府的统一部署，严格落实“三定”方案，调整部门职能和内设机构，实现权责统一，按期完成大部制改革。加快发展非公有制经济。着力加强全民创业精神的培养与教育，进一步浓厚创业、亲商、招商氛围。强化创业培训，积极提供场所、融资、技术等服务，全面落实工商税收优惠、小额担保贷款、资金补贴等政策，鼓励和扶持更多劳动者，特别是青年劳动者成为创业者。重点支持高校毕业生、失业人员和返乡农民工自主创业，加快实施“百名大学生创业行动”。着力培养和孵化创业型人才，优化人才结构。进一步改善非公有制经济发展环境，突出扶持一批成长型的中小企业。大力开展招商引资。今明两年是我们争取大政策的黄金期。要在利用政策解决好民生、基础设施欠账的基础上，将政策与招商引资上大项目搞好衔接，努力把政策优势转化为招商优势、发展优势。利用经济转型、老工业基地振兴、天保工程延伸和棚户区改造政策，为落户我市的企业争取信贷、财政、土地等政策支持。完善招商引资机制，落实奖励办法，调动社会各界参与招商引资的积极性，强力推进重点项目招商。实行招商部门领导责任制，将招商任务与部门奖惩挂钩。瞄准大商名企，集中推介优势产业、基础设施等重大项目，争取在引进大企业和大品牌上取得新突破。积极扩大对外贸易。巩固欧、美、日等传统市场，大力拓展国外新兴市场，扶持重点企业优化出口结构，扩大出口比重。加力推进对俄经贸合作战略升级，不断扩大和深化与俄方在木材采伐与精深加工、农业和矿产开发、边境旅游等方面的合作，建好帕什科沃木材加工园区。进一步搞好嘉荫口岸建设，创造优良的通关环境，提高口岸的通关效率和过货量。努力创新融资方式。加强与金融机构开展战略合作，争取长期贷款支持。搭建银企合作桥梁，做大中小企业担保资金平台。通过土地收储专户融资、开发林业金融产品等多种方式，破解融资瓶颈。

六、强力推进基础设施建设，打造特色魅力新林都

突出城乡规划的科学引导作用，进一步完善城市基础设施，不断提升城市的功能和品位。以棚户区改造、建设旅游名镇和实施“三供两治一绿”为牵动，实现城镇面貌三年大变化。加强交通建设。围绕公路建设“三年决战”的要求，抓好伊绥高速、黑嘉公路、绥嘉公路等在建工程，开工建设前嫩公路伊春段高速路工程，建设农村公路767公里。争取汤嘉铁路和伊春公路客运中心早日开工建设。实施林都大街拓宽、回龙湾大街扩建、青山大街改造等工程。推进“三供两治一绿”工程。全力抓好市供水管网改造二期续建、中心城配水管网、农村饮用水安全、华能热电联产等项目建设，认真做好中心城、铁力市、浩良河镇管道供气前期准备工作，在现有比较成型的小区建设天然气管道。加快嘉荫、带岭等地的污水治理和铁力市垃圾处理工程建设进度，确保早日投产使用。加强城市园林绿化美化和景点景观建设，今年全市绿地覆盖率提高1.5个百分点，人均公共绿地面积增加1.79平方米，力争早日获批“全国绿化模范城市”。面向外地客户，加大房地产市场建设和营销力度，将伊春建成全国闻名的“候鸟型”避暑和养老基地。搞好小城镇建设。以全省建设旅游名镇为契机，加快汤旺河、五营、铁力、嘉荫、朗乡等城镇建设步伐，全面推进城镇供热、供水、环卫等基础设施建设，促进“景”、“镇”协调融合，全力打造各具特色的旅游城镇。推进城市创建。坚持建管结合，进一步加强城市管理。继续巩固“四城联创”成果，高标准开展新一轮市容环境综合整治，加大城市保洁力度，确保国家卫生城年内挂牌。深入开展精神文明创建活动，力争2011年进入国家文明城市行列。

七、深入实施民生改善工程，促进社会和谐稳定

不断改善人民福祉是本届政府的不懈追求。今年的财政支出要从严控制，但对民生的投入只能增加，不能减少。要重点实施八项利民工程，努力办好32件实事。

（一）“两棚一草一房”工程。牢牢抓住国家和省的政策机遇，以“大决战”的气势，强力推进棚户区改造。依法有情做好拆迁安置工作，严格工程质量监管，确保把这一民心工程办成舒心工程、放心工程。

1、投资36亿元，改造棚户区300万平方米，改善6万户群众的住房条件。全面完成伊春区的连片棚户区改造。

2、投资4亿元，新建廉租住房8175户，解决困难群众的住房问题。

3、投资1.25亿元，改造农村泥草房2100户15万平方米。

（二）就业再就业工程。坚持扩大就业与稳定就业“两手抓”，继续实施积极的就业政策，广开就业渠道，全年新增就业4.5万人。

1、投入再就业资金7200万元，为撤场下山人员开发2000个公益岗位，为6万人落实灵活就业社保补贴。

2、投入1000万元，免费开展城乡失业人员就业创业培训。

3、安排小额担保贷款2000万元，扶持大中专毕业生、转业复员军人、外出务工返乡人员、登记失业人员和林改承包户等开展创业。

（三）居民生活保障工程。进一步扩大社会保障覆盖面，强化基金征缴和管理，确保按时足额发放。

1、扩大城镇居民医疗保险覆盖范围，参保率达到90％。

2、提高失业保险金发放标准，每人每月增加30元，县区和市区分别达到310元和350元。

3、增加企业退休人员基本养老金，提高退休待遇；启动铁力市新型农村养老保险试点，逐步扩大范围。

4、筹资4000万元，增加低保补助。

5、投入900万元，足额兑现森工系统老工伤人员待遇。

6、筹资2000万元，启动市社会福利服务中心建设工程。

7、投入600万元，新建3800平方米的儿童福利院。

8、巩固前四轮扶贫解困成果，启动实施第五轮“一帮一”扶贫创业工程。

（四）教育发展工程。继续坚持教育优先发展战略，整合优化教育资源，狠抓教育教学质量，深化教育教学改革，积极改善办学条件，推动各级各类教育均衡发展，促进教育公平。

1、筹资1.82亿元，实施14万平方米校舍安全工程，启动建设市公共实训基地，建成伊春职业学院教学主楼、教师进修学院教学楼，完善市一中和市二中的教学附属设施，搞好南岔铁路中小学等市直学校校舍的维修建设。

2、投入1500万元，更新教育教学设备。

3、提高政企合一区和林业局教师工资标准，达到政府办学水平。

4、多渠道筹资，资助200名普通高中贫困学生和300名考入二表以上本科贫困大学生。

（五）医疗卫生健康工程。深化医疗卫生体制改革，落实基本医疗保障和基本药物制度。加强公共卫生服务体系建设，完善城市社区、农村卫生服务网络，努力满足群众就医需求。加强应急机制与体系管理建设，做好甲型H1N1流感等各类疫情防控工作，提高突发公共卫生事件处置能力。

1、筹资9000万元，改扩建市和县区局15所医院业务用房。为林业中心医院新建1.2万平方米、改造1.6万平方米住院楼，使床位达到850张，争取早日进入“三甲”医院行列；建好市中医院住院处和影像楼。

2、免费为万名妇女进行健康普查，促进妇女健康。

3、为新婚夫妇免费提供婚前医学检查，提高婚后家庭幸福指数和生育质量。

（六）文化体育繁荣工程。实施文化精品工程，打造名牌节目，树立伊春新形象；整合文化信息资源，加强文化服务体系建设；贯彻落实《全民健身条例》，大力开展全民健身运动，提高竞技体育实力，全面备战省第十二届运动会。

1、投资1亿元，启动19万平方米的现代化体育场馆建设。

2、投资4000万元，建设规划面积28.6万平方米的市文化公园，为人民群众健身、休闲、娱乐提供场所。

3、消灭区级图书馆空白点，实现区区都有图书馆的目标。

4、投入3000万元，完成市森林博物馆建设及陈列布展。

（七）便民服务工程。从群众关注的热点、难点问题入手，加强为民服务载体建设，解决影响群众工作生活的突出问题。

1、建设300平方米以上的社区服务中心20个，实现每个县区都有1000平方米以上标准化社区服务中心的目标。

2、投入1200万元，更新公交车辆100台，进一步改善中心城区公交条件。

3、投入1000万元，用于补贴航空公司，争取为林都机场增开3条航线，方便市民出行。

4、全面落实《伊春市物业管理规定》，抓好弃管小区的物业管理试点工作，逐步扩大物业管理覆盖面，提高管理水平。

（八）平安伊春创建工程。加快法治城市建设进程，强化社会治安综合治理基层基础建设，健全治安防范机制，严厉打击各种违法犯罪；高度重视信访工作，不断完善群体性事件预防处置预案和矛盾纠纷调处机制，及时化解社会矛盾；加强安全生产和食品药品安全监管，确保人民群众身体健康和生命安全。

1、改建300平方米的信访服务大厅，方便人民群众表达诉求，越级访、集体访、异常访数量同比明显下降。

2、加强社会治安综合治理，提高人防、物防、技防水平，消灭治安防范盲点，刑事案件综合发案率同比明显下降。

3、加强安全生产工作，杜绝重特大事故，减少一般事故，确保事故起数、伤亡人数、财产损失同比明显下降。

八、切实抓好政府自身建设，提高行政能力和服务水平

坚持埋头苦干，奋发有为，用廉洁高效的实际行动，建设人民满意的“六型”政府。深入解放思想。坚持在保增长、重民生、破解难题上解放思想，不论困难多大、压力多大，都将坚持更大更快更好发展的决心不动摇，善于突破，勇于创新，敢于负责，切实把保增长落实到增速提效上，把解难题转化为创新体制机制上，把保民生体现到老百姓的笑脸上。教育全体公务员全面提高综合素质，善于在经济动荡中学习经济，在金融危机中学习金融，在纷繁复杂的矛盾中提高处理社会矛盾的能力。坚持依法行政。全面落实行政执法责任制，规范各级政府行政权力，重点约束经济领域执法行为。全面落实过错追究制，严格按照法定权限和程序行使职权，坚决查处不作为、乱作为、“中梗阻”和冷、横、硬等问题。注重民主决策。主动自觉地接受人大的法律监督和政协的民主监督，充分发挥群众监督和舆论监督的作用，着力推行政务公开，不断提高政府工作的透明度和公信力。办好市长信箱和市长热线，坚持政府领导与网民在线交流制度，畅通政府与群众沟通渠道，认真听取社情民意，及时为民答疑解惑，帮民所需，解民所难，努力做到让人民满意。提升行政效能。继续开展“百企万民评服务”活动。推行电子政务，办好政府门户网站，建好行政服务中心，继续减少审批项目、前置要件和审批环节，有效解决行政审批“体外循环”问题，构建名符其实的“一站式”审批服务体系。继续加强作风建设，切实提高办事效率、工作质量和干事能力，全面执行政府绩效评估和行政问责制度，促进各项决策部署的有效落实。加强廉政建设。坚持从严治政、精细管理，认真落实党风廉政建设责任制，加强以完善惩治和预防腐败体系为重点的反腐倡廉建设。完善部门预算、政府采购、项目公开招投标等制度，加强对专项资金、重大投资项目和权力部门的审计和监察。切实树立过紧日子思想，突出压缩会议讲话、政府文件、临时机构，从紧从严控制公车配置、公费出国出境和公务消费，确保行政办公经费“零增长”，把有限的财力重点投放到保民生、保发展、保稳定上来。

各位代表、同志们，在全市人民的共同努力下，我市一大批历史遗留问题和民生欠账正在得到有效解决，我们已经站在了伊春快速发展的新起点上。让我们在市委的领导下，坚定信心，同心同德，顽强拼搏，真抓实干，全力推动全市经济社会又好又快、更好更快发展，为实现林都的全面振兴而努力奋斗！
